# Editorial: Non-pharmacological Interventions for Schizophrenia: How Much Can Be Achieved and How?

**DOI:** 10.3389/fpsyg.2016.01289

**Published:** 2016-08-29

**Authors:** Christina Andreou, Steffen Moritz

**Affiliations:** ^1^Department of Psychiatry and Psychotherapy, University Medical Center Hamburg-EppendorfHamburg, Germany; ^2^Center for Gender Research and Early Detection, University of Basel Psychiatric ClinicsBasel, Switzerland

**Keywords:** psychosis, schizophrenia, psychotherapeutic processes, metacognition, neurocognition

For the greatest part of the twentieth century, symptoms of schizophrenia such as delusional beliefs were considered to be “non-understandable,” and attempts to explain and treat these symptoms were predominantly influenced by biological conceptualizations (Mander and Kingdon, 2015). However, insights from behavioral, cognitive and social research as well as societal influences (Mueser et al., 2013; Mander and Kingdon, 2015) have contributed to an increasing appreciation of the importance of cognitive and psychological factors in understanding and treating psychotic symptoms. At the same time, there has been growing discontent with the outcomes achieved through antipsychotic medication alone, especially in terms of functional recovery (Leucht et al., 2009; Jääskeläinen et al., 2013). This, combined with the high reported rates of medication non-adherence (Lieberman et al., 2005), has led to a major boost in the development of non-pharmacological interventions for schizophrenia.

Despite promising results, there is still much controversy regarding the usefulness and applicability of psychological interventions in clinical practice, and there is still little evidence regarding their mechanisms of action. The present Research Topic addresses these issues.

Naturally, an issue dealing with psychological interventions in schizophrenia could not do without the “heavy artillery,” cognitive behavioral therapy. CBT has been one of the first non-pharmacological interventions to be included in treatment guidelines. However, there is still an ongoing debate about its efficacy (McKenna and Kingdon, 2014). Two articles in the present Research Topic contribute to this debate. Peters et al. provide evidence in favor of CBT effectiveness under routine service delivery conditions in a large sample of patients from a challenging catchment area—a very relevant finding for clinical purposes, since everyday clinical practice may differ from clinical studies in many aspects (e.g., patients with comorbidities, variability in therapist availability and/or experience). On the other hand, Mehl et al. deal with the efficacy of clinical studies on CBT for psychosis. The results of their meta-analysis indicate that CBT has a long-lasting positive effect on delusions compared to standard care, but that this effect might be significantly reduced when CBT is compared to other “active” psychological treatments. However, the authors also provide tentative evidence that theory-driven interventions according to an interventionist-causal approach may lead to improved outcomes compared to standard CBT. Thus, treatment outcomes may be improved using more focused interventions based on knowledge of the factors contributing to psychotic symptoms.

Other papers in this issue take up this latter point as well. Three papers focus on variations of metacognitive training (MCT), one of the first interventions to address not delusions *per se*, but rather reasoning biases associated with their emergence and maintenance. Moritz et al. show that an online metacognitive intervention in the context of a cognitive training program can lead to significant changes of the most prominent biases associated with delusions. So et al. provide evidence that an very brief course of metacognitive training can have beneficial effects in patients with psychosis, and that changes in belief flexibility mediate improvement in delusions. Finally, Balzan and Galletly report on two patients refusing antipsychotic medication, in whom individualized metacognitive therapy led to symptom improvement, confirming that psychological interventions may be a viable option in this patient population (cf. Morrison et al., 2014). An interesting aspect of all three above studies is that they describe short, low-cost interventions that are well suited to address problems such as limited resources and cost considerations, which may hamper the dissemination of psychotherapy interventions (Shafran et al., 2009).

Two papers address the processes of improvement rather than determinants of symptoms: Westermann et al. deal with the therapy process and propose that a structured focus on patient motives can improve outcomes of both psychological and pharmacological interventions in patients with psychosis. Menon et al. discuss factors that may affect outcome in group CBT for psychosis and identify an important issue: Despite the wealth of clinical efficacy studies, there is still very little evidence regarding individual factors that may affect treatment success. The authors acknowledge sample size limitations as a cause for this problem and suggest possible solutions.

In reading the above articles, one could think that the factors implicated in symptoms and their improvement act independently and/or in an additive manner. However, the reader should keep in mind that different factors may dynamically interact with one another, leading to complex associations with symptoms. In a very interesting analysis, Hesse et al. confirm that self-concept is important for the development of paranoid delusions, but also show that self-concept in itself may be affected by neurocognitive deficits. Hence, cognitive remediation training might contribute to the stability of long-term symptom outcome, even though it is not thought to have a direct effect on delusions (Wykes et al., 2011). However, cognitive remediation programs themselves are being influenced by the above dynamic interaction concept, moving away from the simple ‘drill-and-practice’ approach: In their opinion paper, Cella et al. summarize evidence suggesting that a metacognitive focus, i.e., promoting awareness of cognitive strengths and weaknesses, boosts the efficacy of cognitive remediation by helping patients develop strategies to overcome neurocognitive deficits. Interestingly, metacognitive awareness itself may be affected by high self-esteem (Cella et al., 2014). This stresses the importance of keeping account of multiple patient characteristics during therapy, however “simple” its actual focus may be.

Several issues remain open: Many authors in this issue highlight the need to consider outcomes other than psychotic symptoms such as depression or well-being. The reader is also reminded that this Research Topic represents only a small snapshot of a fertile research field that includes a number of alternative approaches (to name but a few, see Kurtz and Richardson, 2012 for social cognitive training, Khoury et al., 2013 for mindfulness interventions, and Grácio et al., 2016 for family interventions). The optimistic take-home message is that, although there is still much work to be done in terms of achieving mainstream status, psychological interventions are not only gradually establishing themselves as effective treatments for psychotic symptoms, but are also furthering our understanding of how these symptoms occur.

## Author contributions

CA, SM carried out literature reviews. CA wrote the first draft of this manuscript. SM critically reviewed the manuscript. Both authors have read and approved the final version of the manuscript.

### Conflict of interest statement

The authors declare that the research was conducted in the absence of any commercial or financial relationships that could be construed as a potential conflict of interest.
